# The lysyl oxidase inhibitor β-aminopropionitrile reduces body weight gain and improves the metabolic profile in diet-induced obesity in rats

**DOI:** 10.1242/dmm.020107

**Published:** 2015-06-01

**Authors:** María Miana, María Galán, Ernesto Martínez-Martínez, Saray Varona, Raquel Jurado-López, Belén Bausa-Miranda, Alfonso Antequera, María Luaces, José Martínez-González, Cristina Rodríguez, Victoria Cachofeiro

**Affiliations:** ^1^ Departamento de Fisiología, Facultad de Medicina, Universidad Complutense, Madrid 28040, Spain; ^2^ Instituto de Investigación Sanitaria Gregorio Marañón (IiSGM), Madrid 28007, Spain; ^3^ Centro de Investigación Cardiovascular (CSIC-ICCC), IIB-Sant Pau, Barcelona 08025, Spain; ^4^ Cardiovascular Translational Research, NavarraBiomed (Fundación Miguel Servet), Pamplona 31008, Spain; ^5^ Upper Gastroenterology & Bariatric Surgery Department, Fuenlabrada University Hospital, Madrid 28942, Spain; ^6^ Cardiology Department, Cardiovascular Institute, Hospital Clínico San Carlos, Madrid 28040, Spain

**Keywords:** Lysyl oxidase, Extracellular matrix, Adipose tissue, Fibrosis, Obesity, Insulin resistance

## Abstract

Extracellular matrix (ECM) remodelling of the adipose tissue plays a pivotal role in the pathophysiology of obesity. The lysyl oxidase (LOX) family of amine oxidases, including LOX and LOX-like (LOXL) isoenzymes, controls ECM maturation, and upregulation of LOX activity is essential in fibrosis; however, its involvement in adipose tissue dysfunction in obesity is unclear. In this study, we observed that LOX is the main isoenzyme expressed in human adipose tissue and that its expression is strongly upregulated in samples from obese individuals that had been referred to bariatric surgery. LOX expression was also induced in the adipose tissue from male Wistar rats fed a high-fat diet (HFD). Interestingly, treatment with β-aminopropionitrile (BAPN), a specific and irreversible inhibitor of LOX activity, attenuated the increase in body weight and fat mass that was observed in obese animals and shifted adipocyte size toward smaller adipocytes. BAPN also ameliorated the increase in collagen content that was observed in adipose tissue from obese animals and improved several metabolic parameters – it ameliorated glucose and insulin levels, decreased homeostasis model assessment (HOMA) index and reduced plasma triglyceride levels. Furthermore, in white adipose tissue from obese animals, BAPN prevented the downregulation of adiponectin and glucose transporter 4 (GLUT4), as well as the increase in suppressor of cytokine signaling 3 (SOCS3) and dipeptidyl peptidase 4 (DPP4) levels, triggered by the HFD. Likewise, in the TNFα-induced insulin-resistant 3T3-L1 adipocyte model, BAPN prevented the downregulation of adiponectin and GLUT4 and the increase in SOCS3 levels, and consequently normalised insulin-stimulated glucose uptake. Therefore, our data provide evidence that LOX plays a pathologically relevant role in the metabolic dysfunction induced by obesity and emphasise the interest of novel pharmacological interventions that target adipose tissue fibrosis and LOX activity for the clinical management of this disease.

## Abstract (precis)

**Highlighted Article:** Lysyl oxidase (LOX) could play a role in the metabolic dysfunction induced by obesity, and consequently the inhibition of LOX activity could be a valuable strategy to ameliorate obesity-related metabolic disturbances.

## INTRODUCTION

In recent years, there has been increasing evidence that the extracellular matrix (ECM) plays a key role in adipose tissue development and function, and fibrosis is now recognised as a crucial player in adipose tissue dysfunction in obesity. In fact, early deposition of connective tissue occurs in subcutaneous white adipose tissue after moderate weight gain and further increases with obesity (Mutch et al., 2009; Divoux et al., 2010; Henegar et al., 2008; Alligier et al., 2012). Interestingly, data from animal models of obesity suggest that this abnormal ECM remodelling of white adipose tissue could contribute to local and systemic metabolic alterations (Halberg et al., 2009; Khan et al., 2009).

Fibrosis arises from the imbalance between ECM synthesis and degradation that culminates in an excessive accumulation of ECM components. Lysyl oxidase (LOX) activity is crucially involved in ECM synthesis and processing. To date, five isoenzymes belonging to the LOX family have been identified, including LOX, LOX-like1 (LOXL1), LOXL2, LOXL3 and LOXL4. They are copper-dependent amine oxidases that exhibit a high degree of homology in the C-terminal domain, which contains the catalytic site. LOX, the archetypal member of this family and the best characterised, catalyses the covalent cross-linking of collagens and elastin fibres (Rodríguez et al., 2008a,b). This enzyme oxidises lysine and hydroxylysine residues in these substrates, leading to the synthesis of highly reactive peptidylsemialdehydes that can spontaneously condense to form the intra- and intermolecular covalent cross-linkages that are responsible for ECM stability. The disturbance of LOX activity induces connective tissue abnormalities related to pathological processes, including cardiovascular diseases, as we have previously described (Miana et al., 2011; Orriols et al., 2014; Raposo et al., 2004; Rodriguez et al., 2002, 2009; Stefanon et al., 2013).

Changes in LOX activity could be involved in the disturbance of adipocyte function in obesity. In fact, LOX expression is upregulated in the white adipose tissue from *ob/ob* mice (Halberg et al., 2009), and it is inhibited during adipocyte differentiation (Dimaculangan et al., 1994). Furthermore, LOX participates in the commitment of pluripotent stem cells to the adipocyte lineage (Huang et al., 2009). However, the potential role of LOX activity in human obesity has been poorly characterised. A transcriptomic study revealed an increase in LOX expression in subcutaneous white adipose tissue from obese subjects, but its pathophysiological relevance remains unclear (Henegar et al., 2008). Therefore, the aim of this study was to explore the role of LOX activity in adipose tissue remodelling and in the metabolic disturbances associated with obesity. For this purpose, we have evaluated the impact of β-aminopropionitrile (BAPN), a specific and irreversible inhibitor of LOX activity, in a model of diet-induced obesity.

**TRANSLATIONAL IMPACT**

**Clinical issue**

Obesity is one of the most serious public health challenges of the 21st century. The risk of a number of major diseases, including type 2 diabetes mellitus, ischemic heart disease, ischemic stroke and several common forms of cancers, is dramatically increased in obese individuals. The epidemic proportions achieved by obesity makes it mandatory to reach a deeper understanding of its underlying pathophysiological mechanisms, which could provide novel therapeutic targets. In recent years, fibrosis has been recognised as a crucial player in adipose tissue dysfunction in obesity. Lysyl oxidase (LOX) activity, which governs extracellular matrix maturation, is essential for tissue fibrosis. However, its contribution to adipose tissue dysfunction in obesity has not been clearly established.

**Results**

This study analyses the role of LOX in adipose tissue remodelling by using three experimental systems: adipose tissue samples from obese individuals that had been referred for bariatric surgery (weight loss surgery), an animal model of diet-induced obesity and cell-based studies. The authors demonstrate that LOX is the main lysyl oxidase isoenzyme expressed in human adipose tissue and that it is upregulated in samples from both obese individuals and rats fed a high-fat diet. In obese rats, the inhibition of LOX activity through β-aminopropionitrile (BAPN, a specific inhibitor of LOX activity) reduces adipose tissue fibrosis, partially corrects the adipocyte-size distribution pattern (shifting it toward smaller sizes) and attenuates the increase in body weight and fat mass. Furthermore, LOX inhibition improves multiple metabolic parameters: normalizing glucose, insulin and triglyceride levels and reducing the homeostatic model assessment (HOMA) index – a measure of insulin resistance. Likewise, in these animals, BAPN ameliorates the disturbances in the adipose tissue expression of adiponectin, glucose transporter 4 (GLUT4), suppressor of cytokine signaling 3 (SOCS3) and dipeptidyl peptidase 4, all proteins involved in the control of insulin sensitivity. Finally, BAPN also normalises the insulin-stimulated glucose uptake and protein levels of GLUT4, adiponectin and SOCS3 in the TNFα-induced insulin-resistant 3T3-L1 adipocyte model.

**Implications and future directions**

The results reported in this study demonstrate the upregulation of the adipose tissue expression of LOX in human obesity and provide evidence that LOX inhibition prevents adipose tissue dysfunction, reduces bodyweight gain and improves metabolic disturbances in diet-induced obesity. These findings uncover the pathologically relevant contribution of LOX to the metabolic dysfunction induced by obesity and emphasise the potential of novel pharmacological interventions targeting adipose tissue fibrosis and LOX activity for the clinical management of obesity. Future studies will help to further clarify the mechanisms underlying the beneficial consequences of LOX inhibition in obesity.

## RESULTS

### LOX expression is induced in adipose tissue from morbidly obese patients

**Fig. 1.**
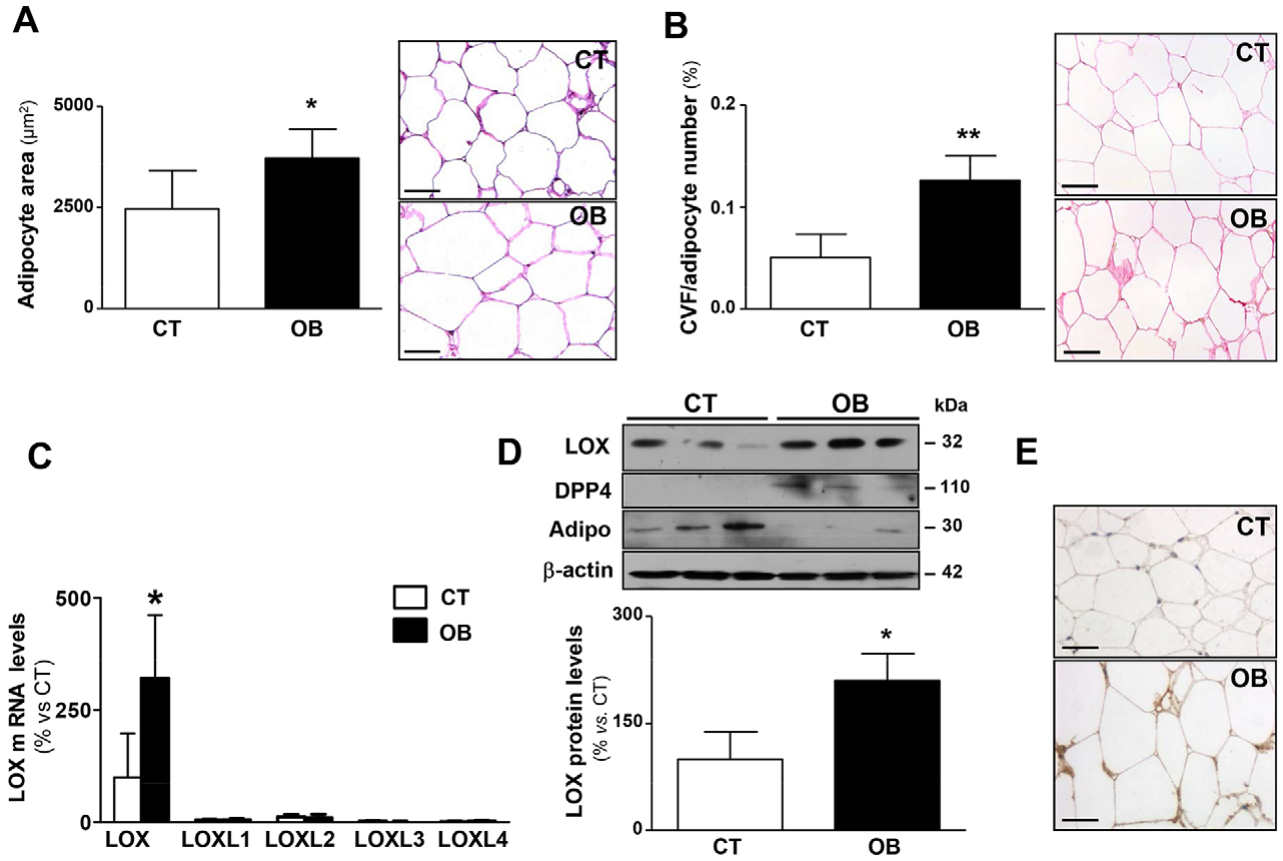
**Adipocyte size, total collagen content and lysyl oxidase expression in visceral adipose tissue from control subjects and morbidly obese individuals.** (A) Quantification of adipocyte area (left) and representative microphotographs of visceral adipose tissue sections from control subjects (CT) and morbidly obese individuals (OB) stained with haematoxylin and eosin and then examined by using light microscopy (magnification 40×; right). (B) Quantification of collagen volume fraction (CVF) normalised for adipocyte number. Right panel shows representative microphotographs of visceral adipose tissue sections from these individuals stained with Picrosirius red that were then examined by using light microscopy (magnification 40×). (C) mRNA levels of lysyl oxidase (LOX) and LOX-like (LOXL) isoenzymes 1, 2, 3 and 4 evaluated using real-time PCR of these samples. Results were normalised to the expression of *18S* RNA. (D) LOX, DPP4 and adiponectin (Adipo) protein levels determined by western blotting. β-actin was analysed as loading control. (E) Representative images of the immunohistochemical analysis for LOX are presented (magnification 40×). Histograms represent the mean±s.d. of five subjects. Scale bars: 50 µm. *P<0.05 and **P<0.01 vs control group.

The expression of LOX and LOXL isoenzymes in visceral adipose tissue was investigated in samples from control individuals and morbidly obese patients. The mean age of the obese group was 40.2±9.5 (range 30-55) vs 36.0±9.5 (range 26-51) years in the normoweight group. The average BMI was 44.6±3.7 (range 41.5-50.9) in the obese group vs 22.7±3.6 (range 16.4-25.0) in the control group (*P*=0.008). No significant differences were observed in the fasting plasma glucose levels between both groups (114.2±26.0 vs 95.8±10.8 mg/dl; *P*=0.344). However, obese patients showed higher insulin plasma levels (486.6±173.1 vs 268.4±138.4 pg/dl; *P*=0.0929), homeostasis model assessment (HOMA) index (3.21±1.49 vs 1.44±0.75; *P*=0.075) and plasma triglyceride levels (169.4±44.8 vs 106.6±24.9 mg/dl; *P*=0.0554) than controls, although neither difference reached statistical significance. As expected, histological analysis of visceral adipose tissue showed an enhanced adipocyte area (cross-sectional area of single adipocytes) (Fig. 1A; *P*=0.05) and an increase in pericellular collagen (Fig. 1B; *P*=0.007) in obese patients as compared with control subjects. Among LOX family members, LOX exhibited the highest expression level in the adipose tissue from control individuals, whereas that of LOXLs was almost negligible. Interestingly, *LOX* mRNA levels were significantly upregulated (about threefold) in the visceral adipose tissue from obese patients compared with that of controls. A consequent increase in LOX protein levels, assessed by western blotting and immunohistochemistry (Fig. 1D,E), was detected in samples from obese subjects. LOX protein levels were correlated with collagen content (r=0.70; *P*=0.0433). As previously described (Kern et al., 2003; Sell et al., 2013), visceral adipose tissue from obese patients showed an increase in dipeptidyl peptidase 4 **(**DPP4) protein levels (220.9±62.6 vs 100.0±10.0 AU; *P*=0.015; Fig. 1D) and a reduction in those of adiponectin (22.7±2.8 vs 100.0±52.4 AU; *P*=0.05; Fig. 1D) compared with normoweight subjects.

### LOX expression is induced in adipose tissue from HFD-fed rats

**Fig. 2.**
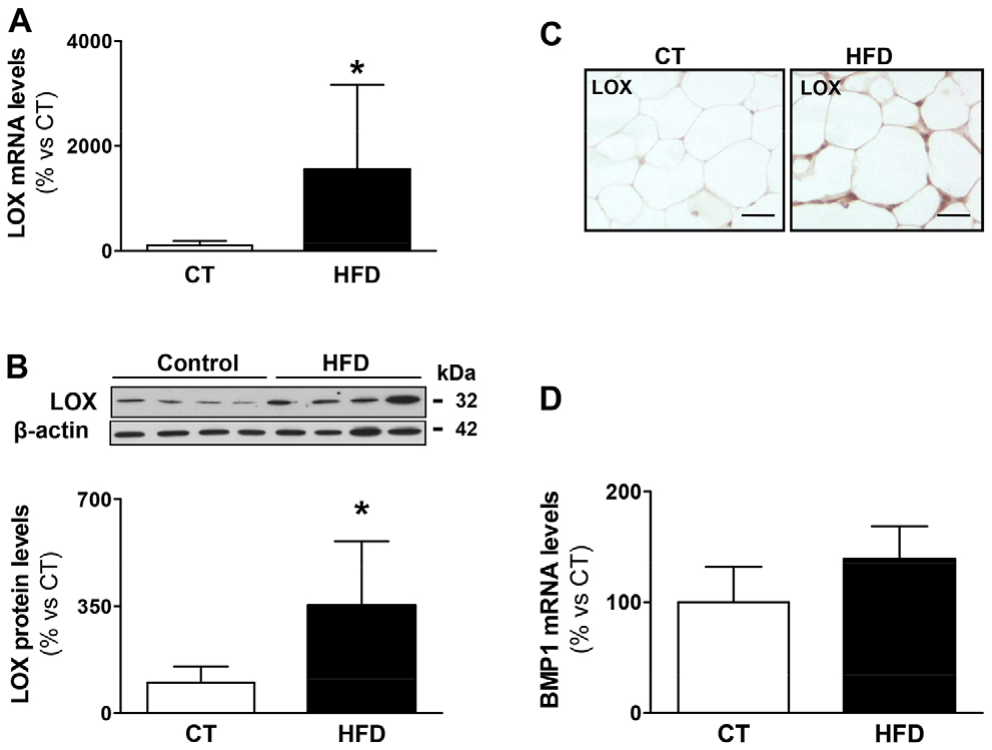
**Expression of LOX and BMP1 in epididymal adipose tissue in control and obese rats.** (A) Lysyl oxidase (LOX) mRNA levels analysed by using real-time PCR of epididymal adipose tissue from control rats fed a normal chow (CT) and rats fed a high-fat diet (HFD). Results were normalised to cyclophilin expression. (B) LOX protein levels determined by western blotting of the same samples described in A. β-actin was analysed as loading control. (C) Representative images of immunostaining of LOX are presented (magnification 40×). (D) Bone morphogenetic protein-1 (BMP1) mRNA levels were quantified by using real-time PCR analyses of epididymal adipose tissue from control rats fed a normal chow (CT) and rats fed a HFD. Expression was normalised to that of cyclophilin. Graphs represent the mean±s.d. of eight animals. Scale bars: 50 µm. *P<0.05 vs control group.

The expression of LOX in white adipose tissue was investigated in an animal model of diet-induced obesity that we have previously described (Martínez-Martínez et al., 2014a,b). As shown in Fig. 2A, *LOX* mRNA levels were increased approximately 15-fold in the epididymal adipose tissue of rats fed a high-fat diet (HFD) compared with control animals fed normal chow. In agreement, LOX protein levels, assessed by western blotting and immunohistochemistry (Fig. 2B,C), were higher in the white adipose tissue from obese animals. A correlation was observed between protein levels of LOX and collagen content (r=0.8389; *P*=0.003). The HFD did not affect the expression of bone morphogenetic protein 1 (BMP1), the enzyme that processes pro-LOX to its active form (Fig. 2D). Furthermore, this dietary intervention did not modify the expression of LOX in brown adipose tissue (data not shown).

### BAPN ameliorates the increase in body weight, adiposity and adipose tissue fibrosis in HFD-fed rats

**Fig. 3.**
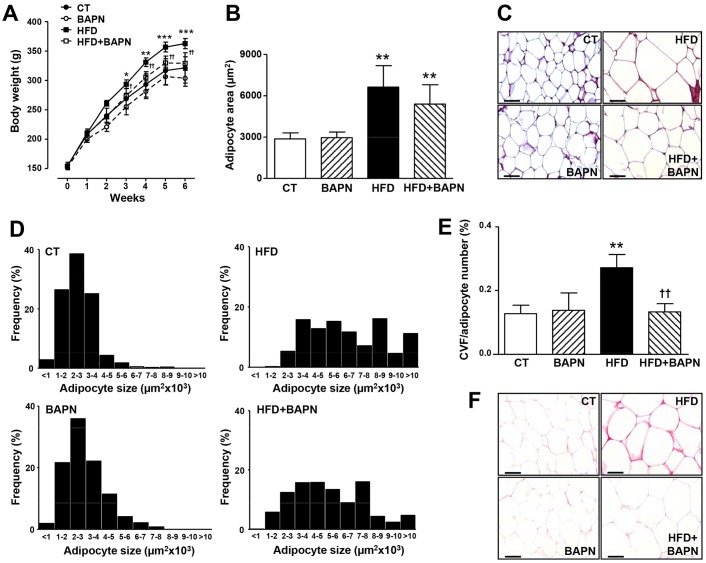
**Effects of lysyl oxidase inhibition on body weight evolution, adipocyte size and distribution and total collagen content in epididymal adipose tissue from control and obese rats.** (A) Body weight evolution along the study. (B) Adipocyte area of epididymal adipose tissue analysed in haematoxylin-eosin-stained sections from control rats fed a normal chow (CT) and rats fed a high-fat diet (HFD) treated with vehicle or with the inhibitor of LOX activity (β-aminopropionitrile, BAPN, 100 mg/kg/day). (C) Representative microphotographs of sections stained with haematoxylin and eosin and then examined by using light microscopy (magnification 40×). (D) Size distribution of adipocytes from epididymal adipose tissue of the animals described in B. (E) Quantification of collagen volume fraction (CVF) normalised for the number of adipocytes analysed in epididymal adipose tissue sections stained with Picrosirius Red. (F) Representative microphotographs of sections stained with Picrosirius Red that were examined by using light microscopy (magnification 40×). Scale bars: 50 µm. Values are mean±s.d. of six to eight animals. *P<0.05, **P<0.01, ***P<0.001 vs control group. ^†^*P*<0.05, ^††^*P*<0.01 vs HFD group.

To investigate whether LOX contributes to the adipose tissue dysfunction associated with obesity, rats subjected to a HFD were treated with BAPN, an irreversible and specific inhibitor of LOX activity. As shown in Fig. 3A, the HFD induced a significant increase in body weight that reached a significant difference from the third week onwards. After three weeks of treatment, BAPN significantly prevented the rise in body weight in HFD rats, but not in animals that were fed a standard diet (Fig. 3A). These differences were sustained until the end of the study (Table 1, Fig. 3A). Similarly, BAPN reduced the increase in the weight of white adipose tissue (both epididymal and lumbar) in obese animals (Table 1) and attenuated their enhanced adiposity (Table 1). It should be noted that the changes in body weight triggered by BAPN in HFD-fed rats were not related to differences in food intake (Table 1).

**Table 1.**
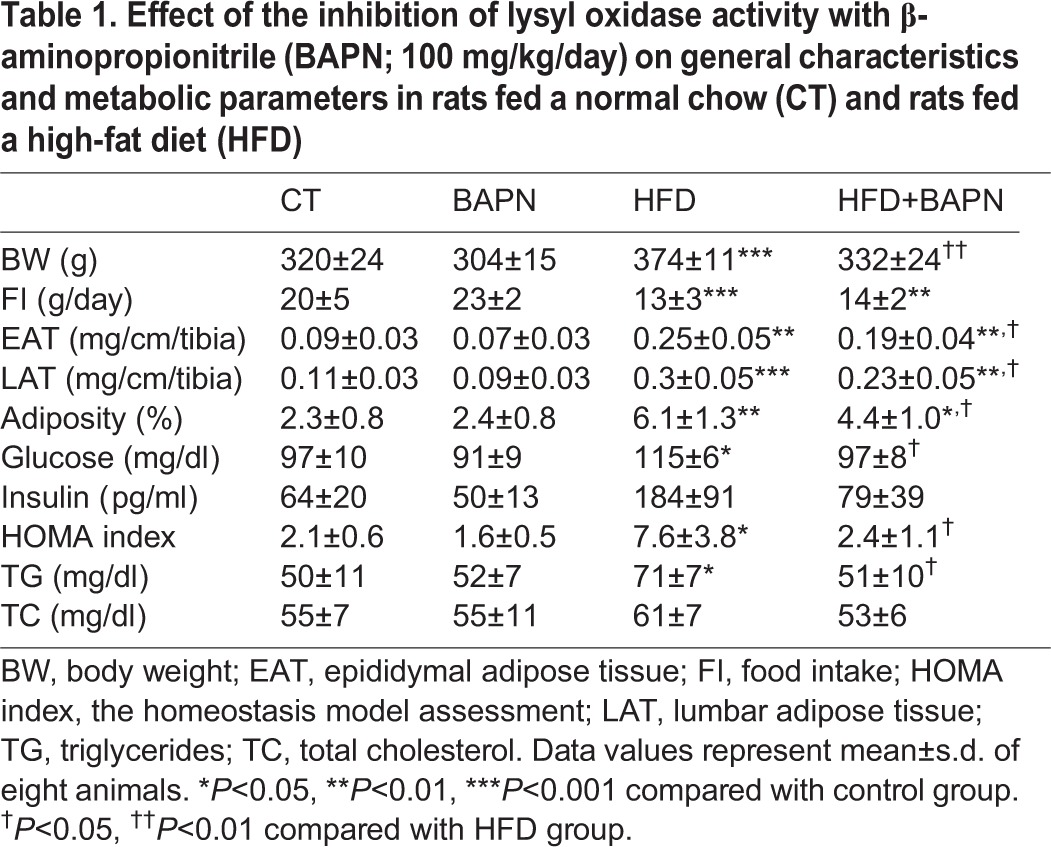
**Effect of the inhibition of lysyl oxidase activity with β-aminopropionitrile (BAPN; 100 mg/kg/day) on general characteristics and metabolic parameters in rats fed a normal chow (CT) and rats fed a high-fat diet (HFD)**

The changes in adipose tissue mass elicited by BAPN in HFD-fed rats prompted us to determine whether LOX inhibition could modulate the adipocyte area. Histological analysis of epididymal adipose tissue revealed an increased adipocyte area in the HFD group. A trend towards an attenuation of this parameter was observed in obese animals that had been treated with BAPN (Fig. 3B,C). Furthermore, a shift toward smaller adipocytes was detected in BAPN-treated obese animals compared with HFD-fed rats (Fig. 3D). Interestingly, LOX inhibition prevented the increase in pericellular collagen content observed in obese rats, as analysed by using Picrosirius red staining (Fig. 3E,F).

### BAPN improves the metabolic alterations observed in obese rats

Next, we examined whether the inhibition of LOX activity could modify metabolic parameters in obese animals. Treatment with BAPN improved fasted glucose and insulin levels and consequently reduced HOMA index in the HFD group (Table 1). LOX inhibition also reduced plasma triglycerides in obese animals, but no significant differences were observed in the total cholesterol levels (Table 1).

### BAPN improves insulin signalling in adipose tissue from obese animals

**Fig. 4.**
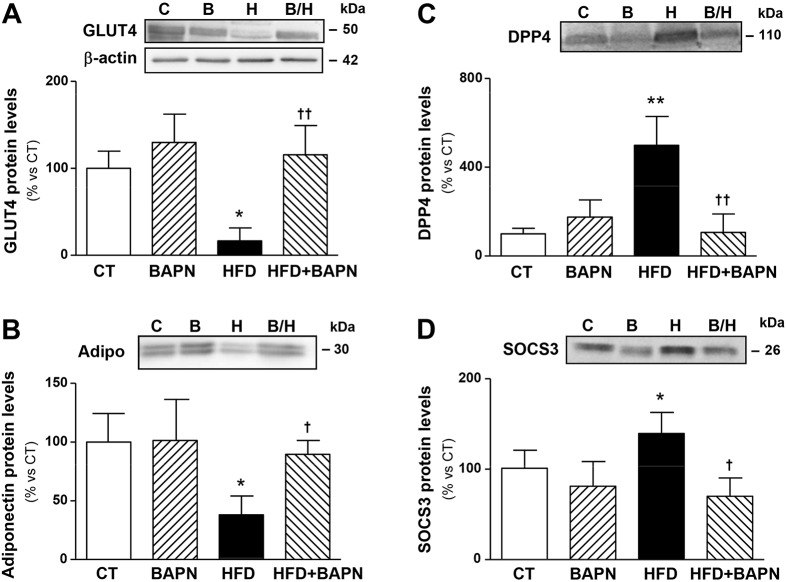
**Effects of lysyl oxidase inhibition on the protein levels of factors involved in the control of insulin sensitivity in epididymal adipose tissue from control and obese rats.** Protein levels of (A) glucose transporter type 4 (GLUT4), (B) adiponectin (Adipo), (C) dipeptidylpeptidase 4 (DPP4) and (D) suppressor of cytokine signaling 3 (SOCS3) in epididymal adipose tissue from control rats fed a normal chow (CT) and rats fed a high-fat diet (HFD) that had been treated with vehicle or with the inhibitor of lysyl oxidase (LOX) activity (β-aminopropionitrile, BAPN, 100 mg/kg/day). Bars on graphs represent the mean±s.d. of six to eight animals, values were normalised to the expression of β-actin. *P<0.05, **P<0.01 vs control group. ^†^*P*<0.05, ^††^*P*<0.01 vs HFD group. ‘C’, control; ‘B’, BAPN; ‘H’, HFD; ‘B/H’, HFD+BAPN.

In order to understand how BAPN improves insulin sensitivity in obese animals, we analysed the levels of proteins involved in the control of insulin sensitivity in epididymal adipose tissue. The reduction in both glucose transporter 4 (GLUT4) and adiponectin expression observed in the HFD group was normalised through the inhibition of LOX activity (Fig. 4A,B). Furthermore, the increase in the protein levels of DPP4 and suppressor of cytokine signaling 3 (SOCS3) triggered by the HFD was completely prevented by BAPN (Fig. 4C,D).

### BAPN normalises the expression of GLUT4 and adiponectin, and improves glucose uptake in an *in vitro* model of insulin resistance

**Fig. 5.**
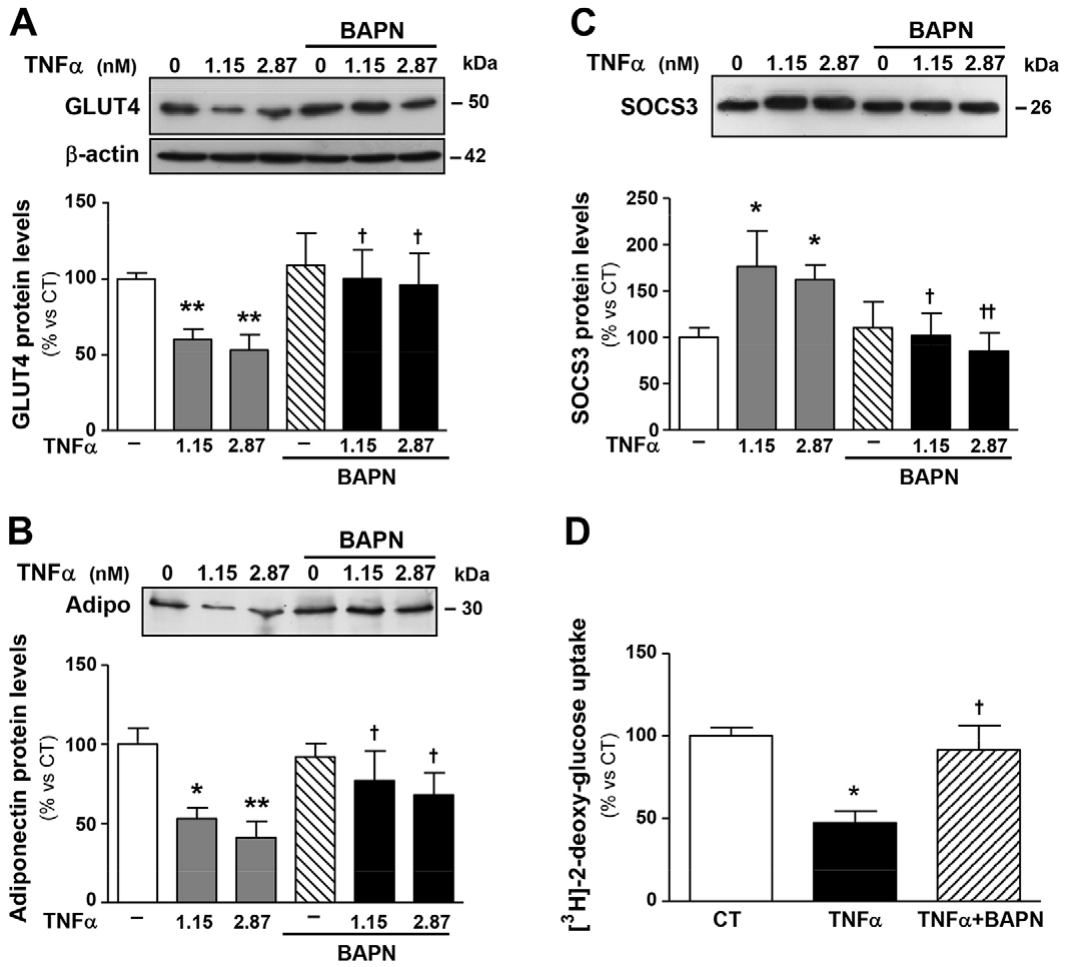
**LOX inhibition influences glucose uptake and the expression of factors involved in the control of insulin sensitivity in an *in vitro* model of insulin resistance.** Protein levels of (A) glucose transporter type 4 (GLUT4), (B) adiponectin (Adipo) and (C) suppressor of cytokine signaling 3 (SOCS3). 3T3-L1 adipocytes were stimulated with or without TNFα (1.15 and 2.87 nM, equivalent to 20 and 50 ng/ml, respectively) for 72 h in the presence of either vehicle or β-aminopropionitrile, an inhibitor of LOX activity (BAPN, 200 µM). Data that were normalised to the expression of β-actin are expressed as mean±s.d. of four assays in arbitrary units. (D) Insulin-stimulated glucose uptake in 3T3-L1 adipocytes. Bars on graphs represent the mean±s.d. of the counts of [^3^H]-2-deoxyglucose normalised to the amount of total protein. Values are mean±s.d. of four assays. *P<0.05, **P<0.01 vs unstimulated cells (TNFα=0); ^†^*P*<0.05, ^††^*P*<0.01 vs cells stimulated with the same concentration of TNFα in the absence of BAPN.

To determine whether BAPN directly affects adipocyte function, we performed *in vitro* experiments in the TNFα-induced insulin resistance model in differentiated 3T3-L1 adipocytes. As shown in Fig. 5 and as previously described (Stephens et al., 1997; Sethi and Hotamisligil, 1999), TNFα reduced expression of GLUT4 and adiponectin and increased SOCS3 protein levels in these cells. Interestingly, these effects were prevented by BAPN (Fig. 5A-C). Accordingly, BAPN normalised the TNFα-induced decrease in insulin-stimulated glucose uptake observed in differentiated 3T3-L1 adipocytes (Fig. 5D).

## DISCUSSION

It is becoming apparent that fibrosis is an essential factor in adipose tissue dysfunction in obesity (Divoux et al., 2010; Halberg et al., 2009; Khan et al., 2009). ECM remodelling impacts on the metabolic function of adipose tissue, and the control of LOX activity, a major player in ECM maturation, could be crucial in this context. The research presented here reveals that LOX is the main lysyl oxidase isoenzyme expressed in adipose tissue and that its expression is upregulated in the adipose tissue of morbidly obese patients and obese rats. More interestingly, we observed that inhibition of LOX activity protects against diet-induced obesity, thereby limiting weight gain, attenuating the disturbances in adipose tissue and improving the insulin resistance observed in obese animals.

Increased expression of ECM components in adipose tissue has been repeatedly reported in murine models of obesity and in obese subjects associated with either body mass index (BMI), insulin sensitivity and/or resistance, and/or inflammation (Abdennour et al., 2014; Divoux et al., 2010; Halberg et al., 2009; Khan et al., 2009; Tam et al., 2012; Walker et al., 2014). A transcriptomic analysis of adipose tissue from obese individuals revealed the pathological relevance of ECM. However, the increase in the expression of LOX and other matrix components observed in these microarray studies was not subsequently validated using other techniques (Henegar et al., 2008). Accordingly, we found that *LOX* mRNA levels are increased in adipose tissue samples from obese individuals, which was confirmed by western blotting and immunohistochemistry. Furthermore, obese individuals exhibited a marked fibrosis of adipose tissue, a pathological process that reduces tissue plasticity and alters adipocyte function (Khan et al., 2009; Virtue and Vidal-Puig, 2010). In agreement with previous reports in the *ob/ob* model (Halberg et al., 2009); we also observed an upregulation of LOX in the adipose tissue of rats fed a HFD that was associated with enhanced pericellular fibrosis. The strong correlation of LOX expression with pericellular fibrosis in the adipose tissue from both humans and rats, as well as the ability of BAPN to limit pericellular fibrosis in HFD-fed animals support the notion that LOX makes a relevant contribution to the fibrotic response in obesity.

LOX and LOXL isoenzymes differ from other copper amino oxidases in their quinone cofactor, the lysine tyrosylquinone (LTQ), which confers particular catalytic properties to these enzymes (Rodriguez et al., 2008b). BAPN is a well-known specific and irreversible inhibitor of LOX activity (Tang et al., 1983; Rodriguez et al., 2008b) that targets the active site of LOX or LOXL isoenzymes. BAPN is a useful tool to study cellular processes involving LOX and LOXL isoenzymes; however, its pharmacological use as a systemic drug is not indicated. It has been observed that prolonged administration of BAPN evokes a weak neurotoxicity and reduces the mechanical strength of bones (Ahsan et al., 1999; Spencer and Schaumburg, 1983; Turecek et al., 2008). Further, lathyrism, a disease characterised by extensive disruption of connective tissue, is caused by the excessive and long-term consumption of certain legumes of the genus *Lathyrus*, which contain high amounts of BAPN and related compounds (Dasler, 1954). Nevertheless, owing to the lack of viable LOX-deficient mice (Maki et al., 2002; Hornstra et al., 2003), this inhibitor has been extensively used in short-term *in vivo* studies to support the involvement of lysyl oxidases in multiple biological processes, including tumour progression and metastasis (Levental et al., 2009; Erler et al., 2006), pulmonary arterial hypertension (Nave et al., 2014) and arterial stiffening (Kothapalli et al., 2012). Similarly, we have observed that BAPN prevents the increase in both body weight and fat-pad weight found in obese animals without affecting food intake. These changes were accompanied by a shift toward smaller adipocytes. Likewise, in mice of an *ob/ob* background, the knockdown of ECM components, such as collagen VI, limited the gain of body weight and reduced the increase in fat mass (Khan et al., 2009). Therefore, the composition and structure of ECM seems to modulate the metabolism of fat tissue in different animal models.

Dysfunction of adipose tissue metabolism has a negative impact on lipid and glucose homeostasis and plays a crucial role in the development of insulin resistance in obesity (Goossens, 2008; Guilherme et al., 2008; Trayhurn, 2013). As in obese individuals, HFD-fed animals also exhibited a decline in insulin sensitivity, as indicated by an increase in HOMA index. This was associated with altered expression of several mediators involved in the control of metabolic functions in the adipose tissue, including adiponectin, GLUT4, SOCS3 and DPP4, as previously described in both humans and animal models (Emanuelli et al., 2001; Kadowaki et al., 2006; Kanatani et al., 2007; Kern et al., 2003; Lamers et al., 2011; Palanivel et al., 2012; Sell et al., 2013; Shepherd and Kahn, 1999; Wang et al., 2000), and with a significant hypertriglyceridemia, a sign frequently observed in the setting of obesity linked to insulin resistance (Eckel, 2011; Taskinen et al., 2011).

More interestingly, we have observed that inhibition of LOX activity attenuates insulin resistance in obese animals. In fact, BAPN reduced insulin and glucose levels and consequently HOMA index, prevented the upregulation of plasma triglycerides and ameliorated the changes in protein expression observed in the adipose tissue from HFD rats. Inhibition of LOX activity reversed the decrease in GLUT4 and adiponectin levels, and the increase in both SOCS3 and DPP4 expression. In view of the low expression of LOXL isoenzymes in human adipose tissue, these data suggest that the upregulation of LOX observed in obesity has multiple consequences for the physiology of adipose tissue. This functional repercussion of LOX upregulation in systemic and adipose tissue metabolism has been suggested previously in a transgenic mouse model that specifically overexpresses HIF-1α in the adipose tissue (Halberg et al., 2009). In agreement with our data, the authors of that study demonstrated that LOX contributes, at least in part, to the metabolic disturbances observed in the model, which were improved by the administration of BAPN. Therefore, our data confirm and extend previous findings on the relevance of LOX in adipocyte function.

The role of LOX in the control of adipocyte function has been poorly characterised. LOX is downregulated during adipocyte differentiation (Dimaculangan et al., 1994), and our data shows that inhibition of LOX impacts adipocyte homeostasis and improves insulin resistance in the well-established model of TNFα-induced insulin resistance. In fact, BAPN normalised the expression of GLUT4, adiponectin and SOCS3, and improved glucose uptake, supporting a direct and more complex role of LOX in adipose tissue physiology. Beyond its role in EMC maturation, LOX exhibits other cellular functions, including the control of epithelial-to-mesenchymal transition, cell proliferation, migration, adhesion and transformation (Huang et al., 2009). The molecular mechanisms underlying the consequences of LOX inhibition in local and systemic metabolism are unknown and, in view of the complex biology of this enzyme, further research is warranted.

In summary, this study demonstrates that LOX could play a relevant role in the metabolic dysfunction induced by obesity and suggests that the inhibition of LOX activity could be a valuable strategy to ameliorate obesity-related metabolic disturbances. These aspects highlight the benefits as well as the complexity of targeting fibrosis as a potential therapeutic intervention in obesity.

## MATERIALS AND METHODS

### Subjects

Visceral adipose tissue was obtained from five individuals that had been referred for bariatric surgery (all women). Inclusion criteria were: age≥18 years and universally accepted indications: long-term obesity (more than 4 years); BMI≥40 despite other weight loss strategies or BMI≥35 in the presence of obesity-related comorbidities (diabetes mellitus, obesity hypoventilation syndrome, obstructive sleep apnoea syndrome and hypertension). Exclusion criteria were: age>60 years and unacceptable surgical risk. Visceral adipose tissue was also obtained from five normoweight BMI≤25 individuals that had been referred to non-bariatric surgery who were included as controls (40% women). The BMI was calculated according to the formula: weight (kg)/square of the height (metres). In accordance with institutional guidelines, all individuals gave informed consent. The study was performed in accordance with the Declaration of Helsinki, and the Ethical Committee of the Fuenlabrada University Hospital approved the protocol.

### Animals

Male Wistar rats of 150 g (Harlan Ibérica, Barcelona, Spain) were fed either a HFD (33.5% fat; Harlan Teklad no. TD.03307, Haslett, MI, USA; *n*=16) or a standard diet (3.5% fat; Harlan Teklad no. TD.2014, Haslett, MI, USA; *n*=16) for 6 weeks. Half of the animals of each group received the irreversible inhibitor of LOX activity BAPN (100 mg/kg/day; Sigma-Aldrich, St Louis, MO, USA) in the drinking water for the same period, as previously described (Brasselet et al., 2005). The amount of BAPN effectively taken daily per animal was calculated from the amount of water consumed on a daily basis. Animal weight was periodically controlled to adjust the target dose of BAPN. Food and water intake were determined throughout the experimental period. Animals were fasted the day before euthanasia by anaesthesia with a cocktail of ketamine (Imalgene 1000, 70 mg/kg; intraperitoneal, Merial Laboratorios, Barcelona, Spain) and xilacine (Rompun 2%, 6 mg/kg; Bayer Hispania, Barcelona, Spain). Serum and plasma were collected and abdominal adipose tissue was dissected for further analysis. Adiposity index was calculated as: sum of fat pads/[(body weight-fat pad weight)×100]. The Animal Care and Use Committee of Universidad Complutense de Madrid approved all experimental procedures according to the Spanish Policy for Animal Protection RD53/2013, which meets the European Union Directive 2010/63/UE.

### Real-time PCR

Total RNA was isolated using Ultraspec™ (Biotecx, Houston, TX, USA) and was reverse transcribed into cDNA using the High Capacity cDNA Reverse Transcription kit (Applied Biosystems, Thermo Fisher Scientific Inc, Waltham, MA, USA). Quantification of mRNA levels was performed by using real-time PCR and an ABI PRISM 7900HT sequence detection system (Applied Biosystems, Thermo Fisher Scientific Inc, Waltham, MA, USA) and TaqMan™ gene expression assays-on-demand (Applied Biosystems, Thermo Fisher Scientific Inc, Waltham, MA, USA) for rat (Rn00566984_m1; RefSeq: NM_017061.2) and human LOX (Hs00184700_m1; RefSeq:NM_002317.5), human LOXL1 (Hs00173746_m1; RefSeq: NM_005576.2), human LOXL2 (Hs00158757_m1; RefSeq: NM_002318.2), human LOXL3 (Hs00261671_m1; RefSeq: NM_032603.2) and human LOXL4 (Hs00260059_m1; RefSeq: NM_032211.6). Rat *BMP1* mRNA was determined by using real-time PCR with SYBR Green PCR Master mix (Applied Biosystems, Thermo Fisher Scientific Inc, Waltham, MA, USA) and the following primers (www.Biomers.net, Donau, Germany): 5′-GTCCTTCACGACAACAAAC-3′ and 5′-GGGTACTTGTCAGGCCAGTT-3′ (positions 2331 to 2434 corresponding to RefSeq: NM_031323.1; amplicon length: 103). *18S* rRNA (4319413E; RefSeq: X03205.1) was used as an endogenous control for human samples. Results from rat samples were normalised according to expression of adenine phosphoribosyltransferase (*Aprt*) or cyclophilin using SYBR Green and specific oligonucleotides (www.Biomers.net, Donau, Germany) as follows: rat Aprt 5′-CGGGCGTGCTGTTCAGGGAT-3′ and 5′-TCAGGTGACCGGCCAGGAGG-3′ (positions 92 to 309 corresponding to RefSeq: L04970.1; amplicon length: 217); rat cyclophilin 5′-TCACCAGGGGAGATGGCACAGG-3′ and 5′-CCATGCTCACCCATCCGGGC-3′ (positions 319 to 419 corresponding to RefSeq: NM_022536.2; amplicon length: 101). Similar results were obtained after normalisation to both housekeeping genes. The ΔΔC_t_ (or ΔΔC_q_ following MIQE nomenclature) method was used for normalisation. In order to appropriately apply this method, we checked that both the target and the reference genes were amplified with comparable efficiencies (>95%, calculated on the basis of the slope of calibration curves). Each sample was amplified in duplicate.

### Morphological and histological evaluation

Visceral and epididymal adipose tissue samples were dehydrated, embedded in paraffin and cut into 5-μm-thick sections. Sections were stained with Picrosirius Red in order to detect collagen fibres. The area of pericellular fibrosis was identified as the ratio of collagen deposition to the total tissue area after excluding the vessel area from the region of interest. This value was normalised by the number of adipocytes. Adipocytes (80-100 per subject or animal) with intact cellular membranes were chosen for determination of the cross-sectional area in hematoxylin-eosin stained sections. For each sample, 10-15 fields were analysed using a 40× objective (Leica DM 2000; Leica Camera AG, Wetzlar, Germany) and quantified (Leica Q550 IWB; Leica Camera AG, Wetzlar, Germany). A single researcher that was unaware of the experimental groups performed the analysis.

### Immunohistological evaluation

Sections were deparaffinised in xylene and rehydrated in graded ethanol solutions. After blocking, slides were incubated with antibodies against LOX (Abcam, Cambridge, UK). Afterwards, samples were incubated with a biotinylated secondary antibody (Vector Laboratories Inc, Burlingame, CA, USA), washed and treated with the Vectastain (ABC) avidin-biotin peroxidase complex (Vector Laboratories Inc, Burlingame, CA, USA). Colour was developed using 3,3′-diaminobenzidine (DAB) and sections were counterstained with haematoxylin. Negative controls, in which the primary antibody was omitted, were included to test for non-specific binding (Orriols et al., 2014).

### Cell culture and differentiation

Murine 3T3-L1 preadipocytes were cultured to confluence in Dulbecco's modified Eagle's medium (DMEM; Life Technologies, Thermo Fisher Scientific Inc, Waltham, MA, USA) that was supplemented with 10% (v/v) calf serum (Biological Industries, Kibbutz Beit-Haemek, Israel). At 2 days post-confluence (designated day 0), cells were induced to differentiate with DMEM containing a standard induction cocktail of 10% (v/v) fetal bovine serum (FBS; Biological Industries, Kibbutz Beit-Haemek, Israel), 1 μM dexamethasone, 0.5 mM 3-isobutyl-1-methylxanthine (IBMX) and 100 nM insulin (all from Sigma-Aldrich, St Louis, MO, USA). After 48 h, this medium was replaced with DMEM supplemented with 10% FBS and 100 nM of insulin. This medium was changed every 48 h. As a model of insulin resistance, murine TNFα (20 or 50 ng/ml, equivalent to 1.15 and 2.87 nM, respectively, Sigma-Aldrich, St Louis, MO, USA) was added to the cell culture medium 7 days after the induction of differentiation, when more than 95% of the cells had the morphological and biochemical properties of adipocytes. For treatment with TNFα (72 h), fully differentiated 3T3-L1 adipocytes were treated every 24 h with the cytokine as previously described (Stephens et al., 1997). BAPN (200 µM) was added to TNFα-treated cells during the last 24 h of incubation. This treatment did not induce cytotoxicity, as analysed by using the XTT-based assay for cell viability (Roche Diagnostics, Indianapolis, IN, USA).

### Preparation of whole cell and tissue extracts

3T3-L1 preadipocytes were washed with PBS and cell monolayers were harvested in a non-denaturing buffer containing 150 mM NaCl, 10 mM Tris, pH 7.4, 1 mM EGTA, 1 mM EDTA, 1% Triton X-100, 0.5% Nonidet P-40, 1 mM Na_3_VO_4_, 1 µg/ml leupeptin and 1 mM DTT. Samples were extracted for 30 min on ice and centrifuged at 16,000 ***g*** at 4°C for 15 min. Supernatants were analysed for protein content using the BCA kit (Thermo Fisher Scientific Inc, Waltham, MA, USA). Total protein from epididymal adipose tissue was obtained by homogenisation in lysis buffer and centrifugation for 5 min at 12,700 ***g*** (4°C). The tissue extract was then separated from the fat and cellular debris, and analysed for protein content.

### Western blotting

Cell and tissue lysates were separated using SDS-PAGE and transferred to 0.45-μm polyvinylidene difluoride membranes (Immobilon, Merck Millipore, Darmstadt, Germany). Blots were incubated with antibodies against GLUT4 (Santa Cruz Biotechnology Inc, Heidelberg, Germany), adiponectin (Chemicon, Merck Millipore, Darmstadt, Germany), DDP4 (Abcam, Cambridge, UK), SOCS3 (Cell Signaling Technology Inc, Danvers, MA, USA) and LOX (Abcam, Cambridge, UK). Bound antibodies were detected after incubation with a horseradish peroxidase (HRP)-conjugated IgG and then using the Super Signal West Dura Extended Duration Substrate (Thermo Fisher Scientific Inc, Waltham, MA, USA). Equal loading of protein in each lane was verified by Ponceau staining and by western blotting for β-actin (Sigma).

### Glucose uptake measurements

Fully differentiated 3T3-L1 adipocytes were insulin-deprived and pre-treated with BAPN in the presence or absence of TNFα for 24 h. Adipocytes were serum-deprived for 3 h in DMEM supplemented with 2% of fatty-acid-free bovine serum albumin (BSA) with or without TNFα and BAPN. Serum-free medium was then removed and the cells were washed with 1 ml of Krebs-Ringer-HEPES (KRH) buffer pH 7.4 plus 0.2% BSA. Glucose uptake was initiated with 0.9 ml of KRH buffer containing 100 mM insulin for 30 min followed by the addition of 100 µM 2-deoxy-d-glucose and 1 µCi/ml of [^3^H]-2-deoxy-d-glucose/ml. After 10 min, cells were washed with an ice-cold solution of 50 mM d-glucose in PBS three times. Cells were lysed with a buffer containing 0.5 N NaOH and 0.1% sodium dodecyl sulfate (SDS), and the radioactivity retained by the cell lysate was measured using a liquid scintillation counter (LS 6500, Beckman Coulter, Fullerton, CA, USA). Measurements were made in triplicate and corrected for nonspecific diffusion. Counts of [^3^H]-2-deoxyglucose were normalised to protein levels.

### Statistical analysis

Data are expressed as means and standard deviations. Normality of distributions and homogeneity of the variance were verified by means of either the Kolmogorov–Smirnov or Levene's test. Either Pearson or Spearman correlation analysis was used to examine the association among different variables according to whether they were normally distributed or not, respectively. Differences between two groups were analysed by either unpaired Student's *t*-test test or Mann–Whitney as parametric and non-parametric tests, respectively*.* Specific differences between more groups were analysed using Kruskall–Wallis followed by Dunn's test for non-normal distribution variables. For normal distribution variables, one-way analysis of variance followed by Newman–Keuls or Tamahane tests were used whether the variance was homogenous or not, respectively. Data analysis was performed using the statistical program SPSS version 22.0 (SPSS Inc., Chicago, IL, USA). The predetermined significance level was α=0.05.
